# Human‐Centered Design of a Contextualized Service Delivery Model for Families of Infants With Major Congenital Anomalies in Kenya

**DOI:** 10.1002/bdr2.70014

**Published:** 2026-02-02

**Authors:** Audrey Chepkemoi, Molly McPheron, Violet Naanyu, James G. Carlucci, Caroline Kerich, Winnie Matelong, Harold Kooreman, Megan S. McHenry, Caitlin Bernard, Marylydia Kiano, Roselyn Midiwo, Beverly Musick, Constantin T. Yiannoutsos, Kara Wools‐Kaloustian, Rena C. Patel, Edwin Were, John M. Humphrey, EA‐IeDEA consortium

**Affiliations:** ^1^ Department of Pediatrics Moi Teaching and Referral Hospital Eldoret Kenya; ^2^ Department of Pediatrics Indiana University School of Medicine Indianapolis Indiana USA; ^3^ Department of Sociology, Psychology and Anthropology Moi University School of Arts and Social Science Eldoret Kenya; ^4^ Academic Model Providing Access to Healthcare Eldoret Kenya; ^5^ Department of Biostatistics Indiana University School of Medicine Indianapolis Indiana USA; ^6^ Department of Obstetrics and Gynecology Indiana University School of Medicine Indianapolis Indiana USA; ^7^ Department of Epidemiology and Biostatistics City University of New York School of Public Health and Health Policy New York New York USA; ^8^ Department of Medicine Indiana University School of Medicine Indianapolis Indiana USA; ^9^ Department of Medicine University of Alabama at Birmingham Heersink School of Medicine Birmingham Alabama USA; ^10^ Department of Reproductive Health Moi University School of Medicine Eldoret Kenya

**Keywords:** birth surveillance, caregivers, congenital anomalies, health services, human‐centered design, Kenya, stigma

## Abstract

**Background:**

Congenital anomalies (CAs) are a major cause of childhood mortality and disability in low‐ and middle‐income countries. Our study explored caregiver experiences of infants with major CAs in Kenya and co‐developed interventions using human‐centered design (HCD).

**Methods:**

We conducted a qualitative study at Kenya's second largest referral hospital (August 2023 to January 2024). Thirty‐one caregivers of 23 infants with major CAs completed interviews on experiences and care needs, analyzed thematically using the socio‐ecological model (individual, family, healthcare, and community domains). We conducted three HCD workshops with 19 healthcare providers and 15 caregivers to co‐develop interventions to improve CA services.

**Results:**

Caregivers reported emotional distress, stigma, and financial and geographic barriers to care. Key healthcare challenges included limited antenatal diagnosis, inadequate provider communication, insufficient peer support, and poor access to CA information. Community stigma contributed to parental isolation and distress, though social and spiritual networks offered coping support. Workshop participants identified stigma and fragmented care as critical issues and proposed feasible interventions, including caregiver support groups, dedicated counselors, provider training, integrated community counseling, improved infrastructure, and stronger support networks to enhance person‐centered care.

**Conclusions:**

Engaging caregivers and providers through HCD highlighted major psychosocial and healthcare barriers and generated contextually relevant strategies to improve care for infants with CAs in Kenya. Future research should evaluate the implementation and effects of these interventions on patient‐ and family‐centered outcomes.

## 1 Introduction

Congenital anomalies (CAs) are important causes of child mortality and long‐term disability in low‐ and middle‐income countries (LMIC), particularly as deaths from infectious disease and injuries decline (Anderson et al. 2008; GBD 2019 Diseases and Injuries Collaborators 2020). In LMICs, constrained health resources can limit timely diagnosis of CAs and access to specialized care (Kingau et al. 2015; Leke et al. 2023; Meherali et al. 2020; Seyi‐Olajide et al. 2023). Low socioeconomic status and health literacy, and stigma associated with CAs are further challenges (Kingau et al. 2015; Meherali et al. 2020). In East Africa, caregivers of children with CAs have reported insufficient training and support to manage their complex needs (Dellicour et al. 2013; Nabatanzi et al. 2021). Developing person‐centered care services for infants with CAs and their caregivers in resource‐limited settings requires a deeper understanding of their needs and preferences, as perceived by caregivers and healthcare providers.

CAs are structural or functional abnormalities that develop prenatally and are detectable before or at birth (WHO 2022). They are classified as major or minor, with major CAs defined as having medical, surgical, or cosmetic significance (WHO 2022). Major CAs account for the greatest share of CA‐related mortality and disability and are thus prioritized in CA service delivery (Diseases and Injuries 2020). Common examples identifiable on infant surface examination at birth include neural tube defects, cleft lip, clubfoot, and Down syndrome; internal anomalies, such as heart or organ defects, may go undetected without appropriate diagnostic tools.

Addressing the complex needs of infants with CAs and their families requires a shift toward person‐centered care models that prioritize dignity, respect, and responsiveness to individual needs and preferences (Giusti et al. 2020; WHO 2013). Such models must be grounded in a deep understanding of the lived experiences of affected families and the barriers to service delivery faced by providers. Human‐centered design (HCD) offers a structured, participatory approach to service model development that centers the perspectives of end‐users (patients, families, providers, and health system leaders) to co‐create contextualized, sustainable interventions to achieve person‐centered care (Beres et al. 2019; Tolley 2017; Leung et al. 2020). The objective of our study was to apply a HCD approach to understand the experiences and care needs of caregivers of infants with major CAs and their healthcare providers in Kenya and inform the development of contextually relevant, person‐centered service interventions.

## 2 Materials/Subjects and Methods

### 2.1 Design and Conceptual Framework

**FIGURE 1:**
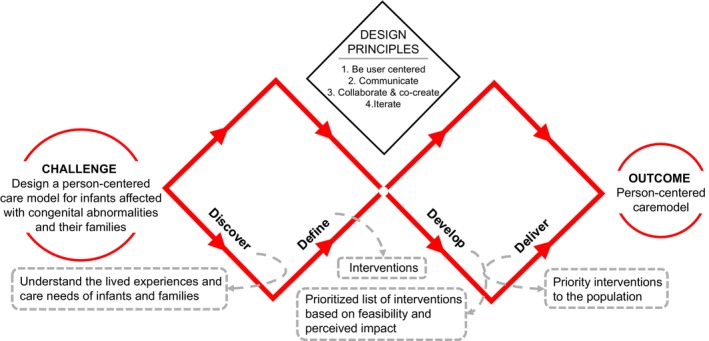
Human‐centered design framework for person‐centered services for infants with major congenital anomalies and their caregivers.

We used the Design Council's Double Diamond framework to guide this qualitative study (Figure 1) (The Design Council 2024). This framework utilizes four phases: discover, define, develop, and deliver. In the *discover* phase, we conducted in‐depth interviews with parents of infants with major CAs exploring their experiences and care needs. For the *define* and *develop* phases, we held three HCD workshops with a subset of interviewed parents and healthcare providers from antenatal, postnatal, and newborn units involved in CA care to define and develop interventions to improve person‐centered services for this population. Individual workshops were held with parents and providers, and the third workshop included both groups.

### 2.2 Setting

The study was conducted at Moi Teaching and Referral Hospital (MTRH) in Eldoret, Kenya, the country's second largest public referral hospital with over 12,000 annual deliveries. MTRH has antenatal and postnatal clinics and a newborn intensive care unit with neonatologists, but no geneticists or genetic counselors. MTRH is the headquarters of the Academic Model for Providing Access to Healthcare (AMPATH), which supports HIV care and treatment at Ministry of Health facilities throughout western Kenya (Kenya Ministry of Health 2022). MTRH is also a participating site in the East Africa International Epidemiology Databases to Evaluate AIDS East Africa (EA‐IeDEA) consortium (Egger et al. 2012).

### 2.3 Recruitment

We recruited parents of infants with CAs participating in the Measuring Adverse Pregnancy and Newborn Congenital Outcomes (MANGO) study within EA‐IeDEA (Humphrey et al. 2025). MANGO is an observational pharmacovigilance study evaluating associations between antiretrovirals and other drug exposures during pregnancy and adverse pregnancy and newborn outcomes, including CAs. The study protocol is available via the WHO Antiretrovirals in pregnancy research toolkit (WHO 2024). MANGO includes a prospective cohort of liveborn infants ≥ 28 weeks gestation at MTRH with major CAs diagnosed on surface exam.

Parents were eligible if enrolled in MANGO, aged ≥ 18 years, fluent in English or Kiswahili, residing within 2 h of MTRH, and delivered a live infant with a major CA at least 6 months before study enrollment (WHO 2022). We used purposive sampling to capture diverse perspectives, including parents of infants with different CA types. We attempted ≥ 3 phone calls to contact each mother. Male partners were recruited during calls with the mother or separately with the mother's consent. Mothers remained eligible regardless of partner participation. Partners of women who had not disclosed their HIV status to their partners were not recruited.

For the HCD workshops, we recruited three groups of CA health service end‐users, all aged ≥ 18 and fluent in English or Kiswahili. Group 1 included mid‐level providers from the antenatal clinic, maternity ward, and postnatal clinic at MTRH: mentor mothers (i.e., women with HIV who are peer counselors for pregnant/postpartum women), medical officers, nurses, and social workers. Group 2 included physicians (i.e., pediatricians, pediatric surgeons, obstetricians/gynecologists, neurosurgeons). Group 3 included caregivers of infants with CAs. Workshops were facilitated by A.C., a neonatologist at MTRH. All participants provided written informed consent.

### 2.4 Data Collection and Management

Kenyan research assistants experienced in patient‐oriented research and fluent in English and Kiswahili collected participants' sociodemographic data using REDCap enrollment surveys; infants' clinical data were extracted from the MANGO database. Audio recordings from interviews and workshops in Kiswahili were transcribed and translated into English. Transcripts were reviewed for accuracy and de‐identified for analysis. The interview and workshop guides were grounded in the socio‐ecological model and developed by study team members, with pilot testing among two mentor mothers and two nurses to ensure clarity and relevance (Tong et al. 2007).

Each 3‐h HCD workshop was audio‐recorded and facilitated by bilingual moderators with HCD expertise. Workshops began with a presentation of *discovery*‐phase findings on the experiences and care needs of families. Participants provided feedback, *defined* potential person‐centered interventions, and discussed hypothesized mechanisms of action. During the *develop* phase, participants evaluated intervention feasibility, identified unmet needs, and prioritized interventions based on projected impact and opportunity costs. Workshops concluded with consensus on next steps for *delivering* prioritized interventions and identifying areas for further research.

### 2.5 Analysis

We conducted thematic analysis of interview transcripts from parents in the *discover* phase using the socio‐ecological model in Figure 3 (Sallis et al. 2008). The individual domain included maternal health beliefs, stigma, and socioeconomic factors; the family domain captured the influence and experiences of partners and other children on care access and outcomes; the healthcare domain encompassed facility environments, leadership, and organizational culture; and the community domain addressed sociocultural norms, policies and values. Two trained analysts (R.M., V.N.) independently conducted axial and selective coding. Axial coding involved organizing initial codes into categories by identifying relationships among concepts, while selective coding focused on refining and integrating these categories around central themes to develop overarching insights (Strauss and Corbin 1998). Themes were organized by domain, with illustrative quotes compared by participant gender and infant CA type. NVivo 12 (QSR International, Melbourne, Australia) was used for data management and coding. For the workshops, A.C. and R.M. used rapid thematic analysis to synthesize participant insights in real time, displaying key themes on wall charts (Taylor et al. 2018). Formal thematic analysis of workshop transcripts followed the same approach as the interviews and was conducted by A.C. and J.M.H. Our reporting follows the SQUIRE framework.

### 2.6 Ethics Statement

The study was approved by Moi University/MTRH institutional Research and Ethics Committee in Kenya (#0004301), Indiana University Institutional Review Board in the United States (#16976), and Kenya National Commission for Science, Technology and Innovation (#NACOSTI/P/24/32597).

## 3 Results

### 3.1 In‐Depth Interviews With Caregivers

**FIGURE 2:**
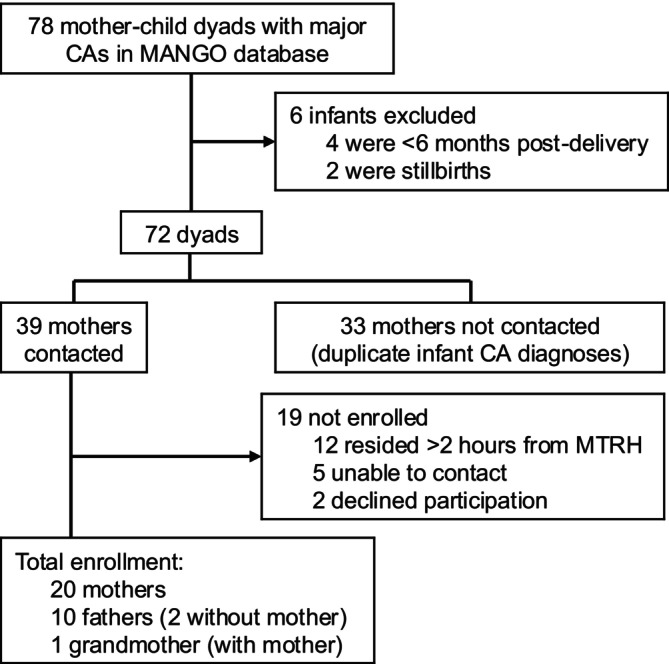
Recruitment flow diagram. CA, congenital anomaly.

From August 2023 to January 2024, 78 mother–infant dyads with major CAs enrolled in the MANGO study were identified (Figure 2). Six dyads were excluded (four infants were < 6 months post‐delivery; two were stillborn), leaving 72 potentially eligible dyads. Contact was attempted for 39; 19 were excluded (12 due to residence > 2 h from MTRH), and 20 dyads enrolled. Additionally, 10 fathers (three with female partner; seven without) and one grandmother were recruited; the grandmother participated at the request of a participating mother (Table 1). In total, 23 infants with major CAs were represented by the participating parents, including one set of twins (Table 2). Four infants died before recruitment. Median infant age at enrollment was 23 months (IQR 15–29), and nine (39%) underwent surgery for CA management.

**TABLE 1:** Characteristics of mother and father caregivers (*n* = 30).

Characteristics	Mothers, *N* = 20	Fathers, *N* = 10
	*N* (%)	*N* (%)
Age in years		
20–29	8 (40)	4 (40)
30–39	10 (50)	5 (50)
≥ 40	2 (10)	1 (10)
County of residence		
Uasin Gishu	17 (85)	8 (80)
Nandi	2 (10)	2 (20)
Kakamega	1 (5)	0 (5)
Employment status		
Formal employment	0 (0)	4 (40)
Self employed	4 (20)	2 (20)
Casual laborer	2 (10)	3 (30)
Farmer	2 (10)	1 (10)
Unemployed	12 (60)	0 (0)
Highest level of completed education		
Primary school	3 (15)	2 (20)
Secondary school	13 (65)	6 (60)
Tertiary school	4 (20)	2 (20)
Number of children		
1	6 (30)	—
2 or more	14 (70)	—
Transferred to MTRH from another facility for delivery	4 (20)	—
Caesarian section delivery	8 (40)	—
Living with HIV	3 (15)	—

Abbreviation: MTRH, Moi Teaching and Referral Hospital.

**TABLE 2:** Characteristics of children with major congenital anomalies.

Characteristics	*N* = 23 (*n* %)
Male sex	13 (57)
< 37 weeks gestation at delivery	4 (17)
Birth weight < 2.5 kg	6 (26)
Deceased by time of recruitment	4 (17)
Months of age at recruitment, median (IQR)^a^	23 (15–29)
Major congenital anomaly	
Talipes equinovarus	4 (17)
Cleft lip and cleft palate	4 (17)
Down's syndrome	2 (9)
Spina bifida, hydrocephalus, talipes equinovarus	2 (9)
Hydrocephalus	2 (9)
Abdominal wall defect	2 (9)
Hypospadias	2 (9)
Spina bifida	1 (4)
Spina bifida, hydrocephalus	1 (4)
Cleft lip	1 (4)
Undiagnosed genetic syndrome	1 (4)
Disorder of sexual differentiation	1 (4)
Surgery to treat congenital anomaly	9 (39)

Abbreviations: IQR, interquartile range; kg, kilogram.

^a^ Among *n* = 19 alive at time of recruitment.

**FIGURE 3:**
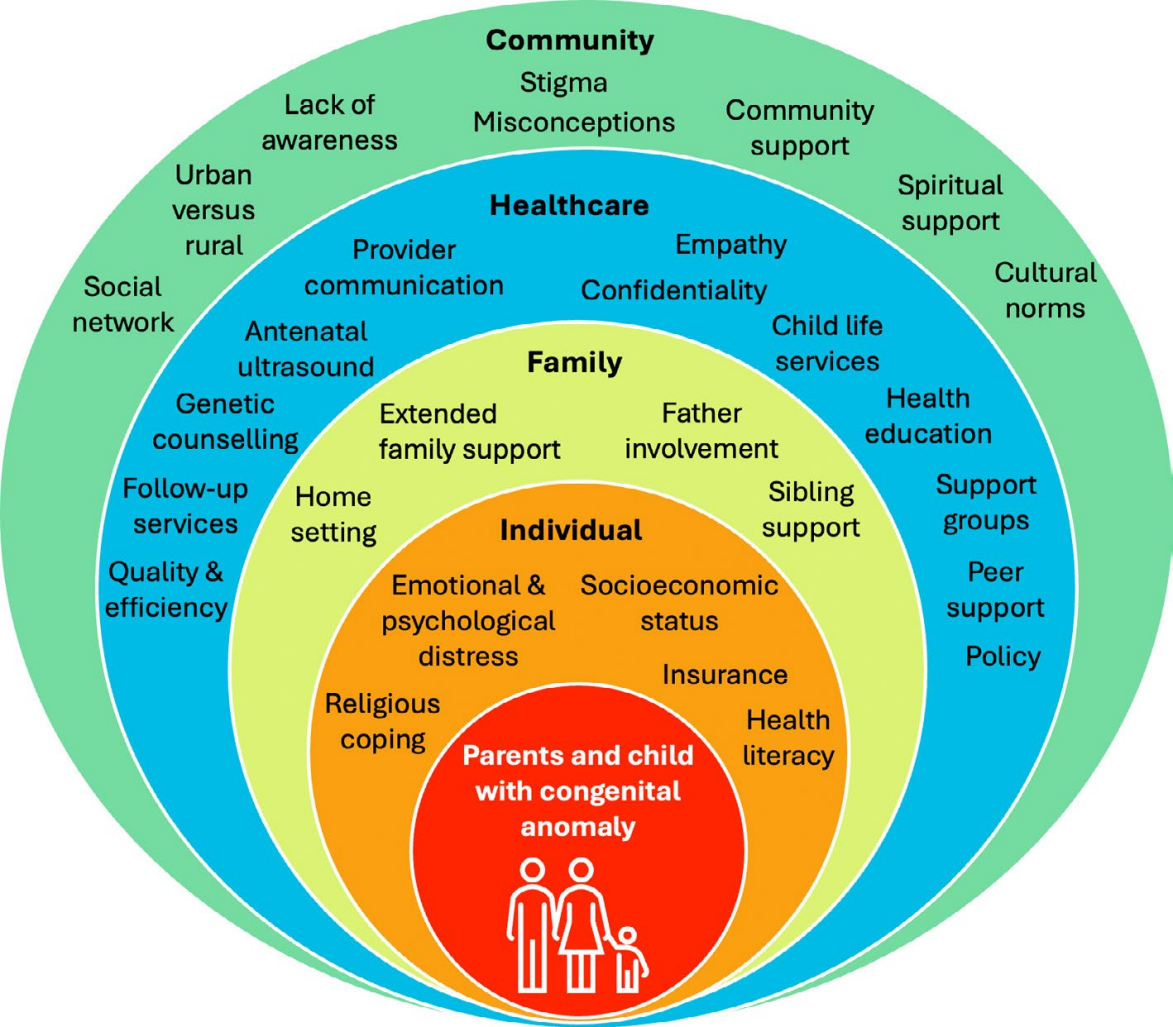
Socio‐ecological factors influencing experiences and care needs of children with major CAs and their caregivers. This figure is adapted from the socio‐ecological model by Sallis et al. (2008).

Nineteen providers were enrolled (100% participation, 74% female), including mentor mothers, social workers, nurses, medical officers, and physicians (Table 3). Caregiver interview themes are presented by socio‐ecological domain, with respondent and infant CA diagnosis in parentheses after each quote (Figure 3).

**TABLE 3:** Characteristics of healthcare providers.

Characteristics	*N* = 19 (*n*, %)
Sex	
Male	5 (26)
Female	14 (74)
Highest level of completed education	
High school	1 (5)
College	4 (21)
University	14 (74)
Occupation	
Peer mentor mother	2 (11)
Social worker	4 (21)
Nurse	7 (37)
Medical officer	1 (5)
Medical specialist^a^	5 (26)
Years in practice^b^	
< 5	2 (11)
5–10	4 (22)
11–20	11 (61)
> 20	1 (6)
Area of practice	
Newborn unit	6 (32)
Pediatric ward or clinic	3 (16)
Obstetrics ward or clinic	8 (42)
Surgery	2 (11)

^a^ Includes pediatrician (*n* = 1), obstetrician/gynecologist (*n* = 2), pediatric surgeon (*n* = 1), neurosurgeon (*n* = 1).

^b^ Among 18 participants.

### 3.2 Individual Domain

The psychological and emotional impact of receiving a CA diagnosis was a major theme. Emotions ranged from fear, shock, and anxiety to passive acceptance as God's will. One caregiver said, “I almost had a heart attack and I was very shocked. I used to arrive to the hospital sweating [when the child was in the newborn intensive care unit]; at times I forgot what I was coming to do” (mother, hydrocephalus). These strong emotions made some parents consider abandoning their child, saying “I was shocked. Even when the child was taken to {newborn unit}, I was about to leave the baby and go away” (mother, spina bifida). Several mothers reported partner abandonment or denial of fatherhood, with one sharing “The father denied the child … to date I don't know where he is” (mother, hydrocephalus). Religious faith also influenced parental acceptance. One said, “I asked God why you have let this child be like this. If you have decided he be that way, I will accept it as he is” (father, cleft lip).

Poverty and distance to health facilities hindered care access. A caregiver of a child needing occupational therapy said, “I do {occupational therapy for the child} myself at home, for I don't have transport” (mother, hydrocephalus). Another, whose child stopped attending clinic explained, “I had to stop due to financial limitations,” adding, “I wish they could find a way to make it a free service,” and suggested to “Build therapy centers in the interior villages to ease the transportation process. If services were available at home, this child would have continued with therapy” (mother, spina bifida).

### 3.3 Family Domain

A child's CA diagnosis affected siblings, who helped support the child while also experiencing distress themselves. One mother said, “My 8‐year‐old is still scared, and he has not been connected with these babies” (mother, cleft lip/palate). Others described sibling involvement in caregiving: “They feed and play with him. There was a time I stopped coming to therapy; they did exercises with him” (mother, spina bifida). Another added, “They teach him how to speak and pronounce words” (mother, cleft lip).

Mothers emphasized fathers' key role in providing emotional, physical, and financial support, saying “He stays with him, feeding him … he plays with him” (mother, cleft lip), and “He does help me … he takes care of school fees” (mother, talipes). Experiences with extended family support varied from help with chores, feeding, and finances to complete lack of involvement. Many parents described feelings of isolation and a sense of personal responsibility, saying “Families now, everybody with their own life. No one can look out for the other” (mother, disorder of sexual differentiation), and “I didn't expect anyone's help. This is my burden I carry it myself” (father, hydrocephalus).

### 3.4 Healthcare Domain

Participants emphasized the importance of early diagnosis via antenatal ultrasound to support care planning and caregiver acceptance. Delayed diagnosis complicated decisions regarding pregnancy termination and referrals, saying “If the child is diagnosed early, you can get help early” (mother, exomphalos), and “If I knew before, I could have aborted” (mother, spina bifida). Families also highlighted the need for clear, compassionate communication from providers to aid acceptance, correct misconceptions, and reduce self‐blame, saying “It is like you have given birth to this child, you have been left to go and struggle on your own … they don't tell you anything” (father, exomphalos). A parent who received the CA diagnosis mid‐cesarean said “The doctor showed me … they both have cleft lip and palate. So, I was shocked … I started crying in the theatre” (mother, hydrocephalus). In contrast, another parent described provider support, saying “I was shocked and very disappointed, the doctor said, ‘This child will be okay’ … some nurses told me, ‘He will be okay’. Then I was encouraged, felt strong, and had hope” (mother, hydrocephalus).

Satisfaction with hospital‐based care varied. Some parents praised inpatient and outpatient services: “When I was in the ward, they took care of me, and when I was at home, they took care of me as well during checkups” (mother, hydrocephalus), and “When she comes home, she comes to tell me that she received great services … it just shows everything is fine” (father, hydrocephalus), and “That's why we didn't even lose hope because of how {good} the services were” (mother, hydrocephalus). Parents also appreciated follow‐up care post‐discharge, saying “Even when I was at home, they used to call me to check on how the baby is doing” (mother, talipes). Others expressed dissatisfaction with service delays and other issues, saying “When the child is ready for the surgery, it should be performed without delay. It is torturing us mentally, physically” (father, cleft lip/palate) and “Sometime the line is long … Sometimes the doctor hasn't arrived or isn't there” (mother, hypospadias). Parents called for improvements, including a private clinic space for children with CAs: “There should be a way of separating people with cleft from others so they have their room and their doctor” (mother, cleft palate). One also emphasized post‐discharge follow‐up: “As a healthcare provider, you should reach out to them and inquire about the child's well‐being” (mother, gastroschisis).

Parents commonly relied on social media and online platforms (TV, Google, YouTube) for information about the CA diagnosis, saying “Most times, I watch the TV programs and you get children with the same problem” (mother, Down syndrome), and “I even started to ‘Google’, I saw children with Down syndrome, then I just accepted” (mother, Down syndrome), and “I checked on the internet, YouTube, looking for videos on how cleft lip and palate goes about, what are the reasons, causes, how it can be repaired” (mother, cleft lip).

Parents emphasized the need for increased health education to support the emotional and physical care of children with CAs, saying “I wish they would advocate in maternity clinics for classes like those for HIV … There should also be classes for abnormalities” (mother with HIV, cleft lip) and “They need to educate people about children with abnormalities” (father, cleft palate). Parents also advocated for peer‐support groups to reduce isolation and share knowledge, sharing “I often feel overwhelmed … I find solace in connecting with other parents” (mother, Down syndrome) and “I normally don't want to talk about my child with people with normal children … If I would get someone with a child with Down syndrome, I would ask them a lot of questions” (mother, Down syndrome), and “I think you can put us on something like WhatsApp … to just help each other” (mother, spina bifida). Participants also called for child life services tailored to the developmental needs of children with CAs.

### 3.5 Community Domain

Perceived and experienced stigma was a prominent theme, contributing to parental isolation, guilt, and, in some cases, child abandonment, noting “Sometimes you are even afraid of taking the baby outside” (mother, hydrocephalus). Another said, “I saw that I would be laughed at by people in the area, and then I came to know that is what made the father deny the child. Till today I don't know where he is” (mother, Down syndrome). Stigma extended to family and peers. One participant described another mother's experience: “She also told me her child made her depressed due to stigmatization. Her own mother even said, ‘I have never seen anyone in this family who birthed a child like this’” (mother, cleft lip/palate). Parents linked stigma to lack of community awareness and misconceptions, saying “There is no help in the community, instead they gossip because they are not aware. They can also stigmatize you” (mother, cleft lip) and “Most are saying it is a curse.” (mother, Down syndrome).

Community support varied, with some parents receiving critical assistance and others feeling abandoned, sharing “I was helped by my neighbors. They would help me buy the diapers and food and the house chores” (mother, gastroschisis). Others reported feeling unsupported: “They don't help; they just see you and they don't do anything” (mother, Down syndrome) and “The community leaves you to struggle” (mother, hypospadias). Spirituality shaped both community perceptions and individual coping, with one parent noting “At the church … at least when you go sing, pray with people even the stress you feel that it is reducing” (father, exomphalos). The importance of health insurance also emerged. One parent advised, “What I would like to tell the other families is they should get {National Health Insurance} or any other insurance that covers health. Yes. Just pay for it, it is important” (father, hydrocephalus).

### 3.6 HCD Workshops

Nineteen providers and 15 caregivers each participated in the workshops, first separately then together in a combined workshop. Reflecting on in‐depth interview findings, providers emphasized the psychological and emotional toll of CAs on families, linking it to mental illness, alcoholism, divorce, and abandonment. As one provider noted, “Most of the parents want to abandon the child.” They described observing parents attributing the CA to medication exposures during pregnancy, witchcraft, food poisoning, infidelity, or past abortions. Providers stressed the importance of addressing stigma through counseling that integrates community beliefs and cultural context: “Incorporate the community's perspective on CAs, their cultural beliefs, and management approaches in counseling sessions”, said one provider. They also acknowledged parental frustration with care delays and called for timelier and coordinated services.

Providers and caregivers proposed multiple interventions to improve person‐centered care for infants with CAs and their families (Table 4). High‐priority interventions included strengthening parent support, care coordination and navigation, and standardized provider training to address care fragmentation, poor quality, and communication gaps. Suggested support strategies included text‐based peer groups (e.g., WhatsApp), educational resources, and counseling. Participants also advocated for longitudinal, family‐centered psychological counseling, modeled on “mentor mother” peer‐support programs used in HIV care during pregnancy and postpartum.

**TABLE 4:** Proposed interventions for person‐centered services for children with congenital anomalies.

Proposed intervention	Mechanisms of action	Feasibility or unmet needs	Relative priority
Individual domain			
Text support group	– Reduce parental feelings of isolation by linking parents – Create forum for information sharing and community building	Group moderator; protections of privacy and confidentiality	High
Educational resources	– List educational websites and other ways to access information about CA diagnoses and management – Improve parental knowledge and skills – Mitigate exposure to harmful or misleading information	Language and cultural barriers; lack of contextually relevant information	High
Family domain			
Family‐based psychosocial support and counseling	– Develop a program that provides support and counseling to the entire family including siblings to help them cope with emotions and reduce fear and confusion	Provider training in family‐based counseling	High
Caregiver education and support group	– Create support group for direct adult caregivers of infants with CAs (e.g., parents, grandparents) to equip them to manage emotional, physical, financial and logistical challenges	Provider training in caregiver‐based counseling	High
Healthcare domain			
Fetal ultrasound	– Early in utero CA diagnosis facilitates counseling, decision making and care planning	Universal fetal ultrasound for all pregnant women; financing	High
Development of CA care protocols	– Enhance quality of care by ensuring comprehensive, standardized care management	Need for MTRH leadership input	High
Multidisciplinary team	– Improve communication and care coordination to improve service quality and efficiency	Available healthcare worker staff and scheduling challenges; limited clinic space; lack of coordinator	High
Peer counselor	– Provide person‐centered counseling to caregivers to help them manage the emotional and psychological impact of the diagnosis, overcome stigma and other barriers to care	Funding	High
Care coordinator	– Ensure linkages between healthcare providers and community health workers to ensure continuity of care; increase parental awareness of health insurance options and linkages to National Health Insurance to improve access to services and reduce out‐of‐pocket costs	Funding	High
Skilled counseling	– Train healthcare providers on effective counseling techniques specifically for infants with CAs – Enable early recognition and management of maladaptive caregiver reactions and behaviors (e.g., mental illness, substance abuse, risk of abandonment)	Clinician expertise to provide training; funding and staff availability to be trained	High
Continuous medical education	– Train health care providers on pathogenesis, diagnosis, management and prognosis of different CAs.	Clinician expertise to provide training; funding and staff availability to be trained	High
Create a clinical database	– Maintain a database of children with CAs to help track retention in care and clinical outcomes over time	Policy to establish benchmarks for care outcomes; determine indicators; privacy and confidentiality	High
Genetic counseling	– Ensure standardized, clear and compassionate communication of CA diagnoses – Answer caregiver questions and deliver accurate information – Provide access to trusted internet resources – Facilitate care decision making	Limited access to provider training, diagnostic testing, and available healthcare personnel	Medium
Counseling checklist	– Develop a checklist to ensure consistent communication of information about the diagnosis, support resources available, and treatment plans prior to diagnosis to enhance parental knowledge and self‐efficacy	Need for policy to inform checklist content; need for patient and MTRH leadership input	Medium
Patient navigator	– Provide person‐centered assistance to help families navigate complex referral schedules and CA treatment programs	Funding	Medium
Enhance documentation in patient‐held medical record	– Include a dedicated section to address CA diagnoses in the patient‐held medical record booklet to enhance documentation and reduce stigma	Policy influences at the national level	Medium
Telemedicine consults	– Offer telemedicine consults (e.g., Project ECHO) for provider‐to‐provider based management of children with CAs at lower‐level facilities to improve reach of specialty services and improve regional care quality	Funding and staff training	Medium
Enhance child life access	– Expand age‐ and disability‐appropriate child life support for children with CAs, especially at lower‐level health facilities to improve child development	Clinic space, funding and staff training	Medium
Private hospital and clinic rooms	– Provide private rooms for parents of infants with CAs in the postnatal ward and postpartum clinic to enhance patient privacy and reduce perceived stigma	Insufficient hospital and clinic space	Low
Increase number of providers	– Increase number of pediatric surgeons and pediatricians to address staff shortages and increase time spent with patients for counseling and care planning	Funding	Low
Dedicated CA clinic	– Implement a dedicated clinic day for multidisciplinary CA care to reduce wait times and enhance care coordination and quality	Clinic space; staff availability	Low
Daycare model	– Introduce daycare for children with CAs and their siblings during clinic visits and post‐discharge to give parents “rest days” to allow time for self‐care and ensuring safe care for their infants with CAs and their siblings	Clinic space, funding and staff training	Low
Community domain			
Home‐based care	– Improve coordination with community health workers and conduct home visits with affected families to improve family preparedness in managing infants with CAs	Funding and coordination with community health volunteers	High
Community engagement and sensitization campaigns	– Launch community sensitization campaigns using social media in rural and urban areas to improve community knowledge about CAs and reduce stigma, and build support networks	Funding and coordination with community health volunteers	Medium

*Note:* Interventions were developed through human‐centered design workshops with providers and caregivers, stratified by socio‐ecologic domain and ranked by participant‐reported priority within each domain.

Abbreviations: CA, congenital anomaly; ECHO, extension for community healthcare outcomes; MTRH, Moi Teaching and Referral Hospital.

To address care fragmentation (i.e., lack of coordination and continuity across multiple providers and services) participants recommended assigning a dedicated care coordinator (e.g., social worker, nurse, or child life specialist) to manage referrals and follow‐up and serve as a liaison between providers and community health workers. Establishing a dedicated clinic day or multidisciplinary clinic for CA care was also proposed to reduce wait times and improve care coordination and quality.

Providers and caregivers noted that CA care often lacked standardization. To improve this, they recommended capacity building through staff education, telemedicine consultations with subspecialists (e.g., geneticists), and increasing the number of surgeons, pediatricians, and neonatologists. Additional suggestions included developing a standardized care checklist, a post‐discharge follow‐up protocol, and a dedicated CA registry to track outcomes and care continuity.

Improving affordability and expanding health insurance coverage were priorities, though implementation was challenging. Strengthening linkages with community health providers was considered essential, with emphasis on adequate remuneration to ensure sustainability. While resource‐intensive, participants also proposed hiring genetic counselors, expanding antenatal ultrasound access, and providing separate inpatient rooms for CA‐affected families. Addressing community‐level challenges, such as stigma and the lack of support networks, was also viewed as important but difficult to implement.

## 4 Discussion

Parents of children with CAs in Kenya reported significant emotional and psychological burdens, including isolation, stigma, and low self‐efficacy and medical knowledge. These findings are consistent with other African studies (Awoyale et al. 2016; Dambi et al. 2015; Kidane et al. 2022; Mazibuko et al. 2022). Parents and providers recommended peer and SMS‐based support groups as feasible, low‐cost interventions to provide emotional support and share knowledge. Platforms like WhatsApp and Facebook could reduce parental feelings of isolation and improve care access, particularly in rural areas (Moges et al. 2023). Similar mobile health (mHealth) interventions have been implemented in resource‐limited settings for HIV, mental health, and maternal‐child health (Bhana et al. 2020; Coleman et al. 2017; Kabongo et al. 2021; Knop et al. 2024; Kruse et al. 2022; Odeny et al. 2014; Ronen et al. 2023; Trude et al. 2021). For instance, a randomized trial in Zambia and Tanzania found WhatsApp groups improved responsive caregiving and caregiver mental health for parents of healthy children (Skeen et al. 2023). With widespread mobile phone use in Kenya, such mHealth approaches could be very adaptable (Kenya National Bureau of Statistics (KNBS), ICF 2023). Further research is needed to determine the optimal platform, facilitation model (e.g., peer parent or nurse moderator), and users' privacy preferences for such interventions.

Caregivers and providers emphasized the need to expand CA services access, noting that children with major CAs require multidisciplinary care. However, financial and transportation barriers limited care access (Commander et al. 2021). Decentralizing services like occupational therapy to lower‐level facilities and cadres of workers, and adopting flexible, family‐centered models that include community health workers, telehealth, home visits, and consistent provider communication could improve continuity of care (Bhutta et al. 2005; Ireland et al. 2019; Leke et al. 2023; Sokunbi et al. 2020).

Caregivers reported major financial strain from medical and transport costs. While health insurance helped offset some expenses, families still faced substantial out‐of‐pocket costs. Similar findings were reported among caregivers in Rwanda, where even well‐insured families struggled with indirect expenses (medications, transport, food) and income loss in caring for children with CAs (Kidane et al. 2022). Participants in our study identified expanding insurance coverage, promoting economic empowerment through microfinance, and increasing affordable care access as high priorities. These challenges also highlighted the need for community sensitization on insurance options and efforts to address economic vulnerability.

Early antenatal ultrasound coupled with structured health education and counseling was proposed to better prepare families for diagnosis and care of children with CAs (Onyambu and Tharamba 2018). Participants recommended specific tools such as a standardized checklist to improve counseling consistency and completeness, convey diagnoses, and connect families with educational resources (websites) and support services (clinics, support groups). Provider education and standardized protocols were proposed to ensure consistent, quality counseling. These low‐cost interventions could facilitate earlier diagnosis and intervention while improving caregiver understanding and communication. Embedding a multidisciplinary psychosocial support team could also offer trauma‐informed support to mitigate psychological distress pre‐ and postnatally (Cole et al. 2025).

A strength of our study is the inclusion of caregivers of children with a variety of major CAs and providers. Potential limitations include social desirability and recall bias among caregivers. To minimize these biases, we used simple, indirect questioning, cross‐checked reported dates and diagnoses with MANGO study records, and assured participants of confidentiality. The perspectives of participants may also differ from nonparticipants, potentially affecting the representativeness of our findings. Additionally, our findings may not be as easily transferrable to rural, lower‐level health facilities or contexts with fewer resources than our study's urban referral facility setting.

In conclusion, engaging caregivers and providers through HCD identified challenges and opportunities to improve person‐centered care for infants with CAs in Kenya. Further research is needed to evaluate the impact of these interventions on patient outcomes, caregiver experiences, and health service delivery.

## Author Contributions

A.C. and J.M.H. designed the study. C.K., W.M., and R.M. collected the data. A.C., J.M.H., H.K., and R.M. analyzed the data. B.M. and H.K. provided data administrative support. C.T.Y., K.W.‐K., R.C.P., and E.W. provided oversight of the study planning and execution. A.C. and J.M.H. wrote the paper with review and editing from all authors.

## Funding

This work was supported by the National Institute of Allergy and Infectious Diseases (NIAID), Eunice Kennedy Shriver National Institute of Child Health and Human Development (NICHD), National Institute on Drug Abuse (NIDA), National Cancer Institute (NCI), National Institute of Mental Health (NIMH), National Institute of Diabetes and Digestive and Kidney Diseases (NIDDK), Fogarty International Center (FIC), National Heart, Lung and Blood Institute (NHLBI), National Institute on Alcohol Abuse and Alcoholism (NIAAA), Intramural Research Program, in accordance with the regulatory requirements of the National Institutes of Health under Grant U01AI069911 East Africa IeDEA Consortium.

## Disclosure

The Double Diamond by the Design Council is licensed under a CC BY 4.0 license, which gives permission to share (copy and redistribute the material in any medium or format for any purpose, even commercially) and adapt (remix, transform, and build upon the material for any purpose) the diagram.

## Ethics Statement

The study was approved by the Moi University/MTRH Institutional Research and Ethics Committee in Kenya (#0004301), the Indiana University Institutional Review Board in the United States (#16976), and the Kenya National Commission for Science, Technology and Innovation (#NACOSTI/P/24/32597).

## Consent

All participants provided written informed consent.

## Conflicts of Interest

The authors declare no conflicts of interest.

## Data Availability

Relevant data from this study are presented in the main manuscript. The identified data are also available upon reasonable request to the Corresponding Author. An open‐source template to use the Double Diamond is published on the website (see www.mural.co/templates/double‐diamond).
